# Effect of Thermosonication on the Nutritional Quality of Lapsi (*Choerospondias axillaris*) Fruit Juice: Application of Advanced Artificial Neural Networks

**DOI:** 10.3390/foods12203723

**Published:** 2023-10-10

**Authors:** Puja Das, Prakash Kumar Nayak, Baskaran Stephen Inbaraj, Minaxi Sharma, Radha krishnan Kesavan, Kandi Sridhar

**Affiliations:** 1 Department of Food Engineering and Technology, Central Institute of Technology Kokrajhar, Kokrajhar 783370, India; pdas12994@gmail.com (P.D.);; 2 Department of Food Science, Fu Jen Catholic University, New Taipei City 242062, Taiwan; sinbaraj@yahoo.com; 3 Department of Applied Biology, University of Science and Technology, Baridua 793101, India; minaxi86sharma@gmail.com; 4 Department of Food Technology, Karpagam Academy of Higher Education (Deemed to be University), Coimbatore 641021, India

**Keywords:** thermosonication, lapsi juice, artificial neural network (ANN), nutritional property, antioxidant activity, microbial inactivation

## Abstract

This study explored the effect of thermosonication on the nutritional properties of lapsi (*Choerospondias axillaris*) fruit juice. The intent of the present investigation was to process lapsi fruit juice using both thermosonication and thermal pasteurisation and to compare the effects of these treatments on the juice’s physicochemical, nutritional, and microbiological qualities. In order to maximise the retention of nutritional properties, enhance juice quality, and boost efficiency, an artificial neural network (ANN) model was also developed to forecast the optimisation of process parameters for the quality of lapsi fruit juice. This study establishes a novel experimental planning method using an ANN to multi-objectively optimise the extraction process and identify the ideal extraction conditions for thermosonication (50, 75, and 100% amplitude at 30, 40, and 50 °C for 15, 30, 45, and 60 min) to augment lapsi juice’s nutritional and microbiological properties by improving certain attributes such as ascorbic acid (AA), antioxidant activity (AOA), total phenolic content (TPC), total flavonoid content (TFC), total plate count, and yeast and mould count (YMC). The maximum values for AA (71.80 ± 0.05 mg/100 mL), AOA (74.60 ± 0.28%), TPC (187.33 ± 0.03 mg gallic acid equivalents [GAE]/mL), TFC (127.27 ± 0.05 mg quercetin equivalents [QE]/mL), total plate count (not detected), and YMC were achieved in thermosonicated lapsi juice (TSLJ) under optimal conditions. For AA and TFC, the optimal conditions were 100% amplitude, 40 °C, and 45 min. For AOA and TPC, the optimal conditions were 100% amplitude, 40 °C, and 60 min, and for YMC, the optimal conditions were 100% amplitude, 50 °C, and 60 min. According to the findings, thermosonicated juices have improved nutritional properties, making them an excellent source of bioactive elements for use in both the food and pharmaceutical sectors. According to this study, ANN has been identified as a valuable tool for predicting the effectiveness of lapsi fruit juice extraction, and the application of thermosonication as an approach for lapsi juice preservation could be a potential successor to thermal pasteurisation. This approach can help to minimise or hinder quality degradation while improving the juice’s functionality.

## 1. Introduction

Fruits and vegetables are vital parts of a nutritious diet. They contain essential minerals, vitamins, and fibre to promote overall wellness and good health. Consuming a variety of fruits and vegetables has been linked to a lower risk of chronic diseases such as stroke, heart disease, and certain cancers [1]. Additionally, the regular consumption of fruits and vegetables could help manage weight, improve digestion, and boost immunity. Northeast India is a treasure trove of underutilised fruits that are not only delicious but also packed with nutrients and health benefits. These fruits are unique to the region and have been traditionally used by the local communities for centuries but remain largely unknown to the outside world [2].

Lapsi (*Choerospondias axillaris*) is a lesser-known fruit native to Bhutan, Nepal, and parts of Northeast India (Sikkim, Arunachal Pradesh, and Assam) [3]. At the beginning of spring, the lapsi tree produces tiny, light-greenish blossoms that are accompanied by tiny, oval fruits around 2–3 centimetres in diameter. When fully ripe, the fruit turns yellow or red and possesses a sour, tart flavour. It has various nutrients (vitamin C, vitamin A, and dietary fibre), possesses numerous health benefits, and is known for its antioxidant, antimicrobial, and anti-inflammatory properties [4]. The fruit has been traditionally used in one of the world’s oldest medical systems, Ayurvedic medicine, India’s traditional health system. The fruit has a unique sour and sweet taste and is used in various culinary preparations, such as pickles, jams, and chutneys.

As lapsi fruits are perishable in nature, it is essential to prevent their loss and promote their use by processing them into value-added products such as fruit juices [5]. The processing of fruits into juices may help to maintain the freshness and long-term viability of the fruits, as well as increase their monetary and nutritional value. It is essential to explore various processing methods and techniques to ensure that the fruits are processed in a safe and sustainable manner while retaining their essential nutrients and sensory properties.

Thermal treatment, such as pasteurisation (PS), is required to ensure the safety and quality of fruit juices [6]. Pasteurisation is a heat-treatment technique that eliminates potentially harmful microorganisms from fruit juices. In addition, by preventing microbial deterioration, it can aid in extending the shelf life of juices [7]. Heat-sensitive micro-nutrients (vitamins) in fruit juices may break down due to the high temperatures used during PS, lowering their nutritional value [8]. Furthermore, the heat may alter the juice’s flavour, aroma, and colour, which may impact its sensory qualities. Alternatives to thermal processing in the manufacturing of fruit juices include non-thermal methods like ultrasonication, cold plasma, irradiation, membrane filtration, ozone treatment, and high-pressure processing (HPP) [9]. While ensuring its safety and extending its shelf life, these methods can aid in maintaining the juice’s nutritional and sensory qualities.

A promising non-thermal technology known as ultrasound (US) treatment can successfully remove microorganisms from fruit juices with little to no effect on nutritional and sensory qualities. Due to its various benefits over conventional thermal processing techniques, this novel method can greatly benefit the fruit juice industry. Thermosonication (TS), a type of ultrasonication method, uses heat and high-frequency sound waves (ultrasound) for food preservation. It involves heating food to a specific temperature (typically between 50 °C and 80 °C) and then exposing it to high-frequency sound waves (typically between 20 kHz and 100 kHz) [10]. The emergence and ensuing disintegration of tiny air bubbles in a liquid is known as cavitation, and it is the main physical mechanism that occurs in thermosonication. These air bubbles form as a result of the passage of ultrasound waves through the liquid. When the air bubbles implode, they raise the surrounding temperature and pressure. This causes localised turbulent flows and vibrations that aid in rupturing the cell walls and releasing the desired compounds [5].

Several researchers have shown the preservative effect of ultrasound on fruit juices, including [11] pomelo juice and [12] kutkura juice. According to Boghossian et al. [13], ultrasound is a well-known method for producing fruit juices of superior quality because it retains more bioactive compounds than thermal methods alone.

An artificial neural network (ANN) is a machine-learning algorithm that draws inspiration from the structure and operations of the human cerebral cortex. ANNs consist of interconnected nodes, often referred to as “neurons,” which play the crucial roles of processing and transmitting data [14]. The steps involved in creating an ANN consist of collecting the data, organising it, training the ANN, testing it in order, analysing its performance, and using it to predict outcomes from new input data. ANNs can be used to create predictive models for complex systems in the context of modelling and optimisation, where conventional mathematical models might not be practical [14]. Based on different processing parameters, they can be used to simulate food processing operations and forecast significant quality attributes. By doing so, the processing conditions can be improved to produce the desired quality attributes while lowering costs and generating less waste. In a variety of industries, including food processing, ANNs provide a versatile and potent tool for modelling and optimisation. They have been used by several researchers to model and optimise process variables for various fruit juices, including cashew apple juice [15], bael fruit juice [16], and red plum juice [17]. It is clear that ANNs offer multiple advantages over other popular optimisation tools, including response surface methodology such as flexibility, non-linearity, robustness, parallel processing, learning and adaptation, lack of prior knowledge requirements, and generalisation.

To date, there have been no reports on the use of thermosonication for the processing lapsi fruit juice. Thus, the objective of this study was to utilise both thermosonication and thermal pasteurisation to process lapsi fruit juice and to assess the resulting influence on the physicochemical, nutritional, and microbiological properties of the juice. Additionally, an ANN model was created to forecast the optimisation of process parameters for the quality of lapsi fruit juice for maximum retention of nutritional properties, improvement of juice quality, and increased efficiency.

## 2. Materials and Methods

### 2.1. Lapsi (Choerospondias axillaris) Fruit Sample

Mature and flawless lapsi fruits, freshly harvested from a garden in the vicinity of Ranipool, Sikkim, were carefully collected and transferred using an insulated container (sterile ice box maintaining 4 °C) to the laboratory of the Department of Food Engineering and Technology, CITK, Kokrajhar. The fruits were removed from their stalks, washed in water, allowed to air dry, and sliced into small pieces before the juice was extracted.

### 2.2. Chemicals

The chemicals utilised in this study were obtained from two different sources, namely Bengaluru-based Sigma Aldrich and Mumbai-based HiMedia Chemicals, nestled in India. Additionally, the chemicals utilised to determine microbiological properties, including potato dextrose agar, total plate count agar, and peptone water, were acquired from MERCK Group.

### 2.3. Juice Preparation

Slices of lapsi fruit (approximately 2–3 cm in size) were crushed using a domestic grinding device (mixer). After that, Whatman number one filter paper was used to eliminate any remaining impurities and clarify the juice. Three separate treatment groups of the juice were established as follows: the raw juice (control), the pasteurised lapsi juice (PSLJ), and the thermosonicated lapsi juice (TSLJ). All juice samples were processed immediately after their separation on the same day.

### 2.4. Pasteurisation of the Juices

Lapsi fruit juices were thermally treated in an experimental establishment employing a vertical pasteuriser at a temperature of 90 °C for a duration of 60 s [12]. After pasteurisation, the samples were cooled in an ice bath. The chosen pasteurisation conditions were aimed to mimic the industrial processing of juices, which typically involves using temperatures ranging from 90 to 95 °C for about 15 to 60 s to reduce the number of microorganisms up to 5 logs. SJ was treated at 90 °C for 60 s with PS.

### 2.5. Thermosonication of the Juices

**Figure 1:**
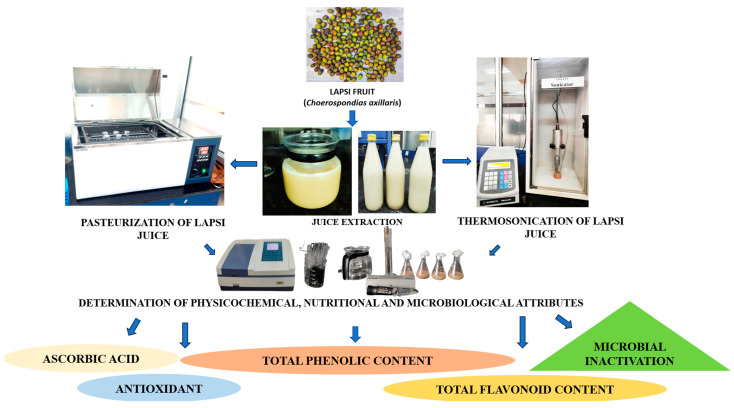
Processing of lapsi fruit juice and studied parameters.

Thermosonication treatments on juice samples were conducted using an ultrasonicator probe (Sonics and Materials, Inc., Newton, CT, USA) at various temperatures (30, 40, and 50 °C) with 750 W full power [18]. Juice samples (100 mL) were treated at intervals of 15, 30, 45, and 60 min at three different amplitudes (50%, 75%, and 100%) [19,20]. The sonication was conducted under dark conditions to avoid any interaction with light. After sonication, the juice samples were transferred to sterilised containers and stored at 4 °C until further analysis. The processing of lapsi fruit juice is represented in Figure 1.

### 2.6. Determination of Physicochemical Properties

#### 2.6.1. pH, Total Soluble Solids, and Titratable Acidity

The pH levels of both control and treated lapsi juice samples were measured at 25 ± 1 °C applying a digital pH meter [21]. The concentration of total soluble solids (TSSs) of lapsi juice was analysed by employing a refractometer, and the results were reported in the form of Brix degrees (°Brix) [22]. The titratable acid concentration (TA) of both control and treated lapsi juice samples was assessed using the AOAC [21] method. The results were expressed as a citric acid percentage [12]. The results for each sample were taken in triplicate and an average was taken for every analysis.

#### 2.6.2. Cloudiness and Browning Index

Centrifugation was carried out using 5 mL samples of both treated and untreated LJ at room temperature for estimating the cloudiness (CI) and browning index (BI). The centrifugation was performed using a centrifuge (Remi C-24BL, Remi Elektrotechnik Ltd., Mumbai, India) spinning at 6000 rpm for a duration of 10 min. After the centrifugation process, the supernatant was utilised to determine CI and BI. To assess the degree of cloudiness, the absorbance was measured at 660 nm using a UV-Vis spectrophotometer (PerkinElmer Lambda 35 UV/VIS, PerkinElmer, Inc., Waltham, MA, USA) [23]. In order to determine the browning index of both treated and untreated LJ, 5 mL of supernatant was mixed with 5 mL of ethanol. The mixture was thoroughly combined, and its absorbance at a wavelength of 420 nm was subsequently measured using a UV-Vis spectrophotometer [23].

### 2.7. Determination of Functional Properties

#### 2.7.1. Ascorbic Acid Content

The level of ascorbic acid content (AA) in LJ samples was measured by employing the iodine titration method [24]. Soluble starch (0.25 g) was dissolved in a 100 mL conical flask with 50 mL of near-boiling water. After cooling, a separate mixture of potassium iodide (2 g) and iodine (1.3 g) was dissolved in distilled water and transferred to a 1 L volumetric flask. Samples (raw, PSLJ, and TSLJ) were diluted with distilled water (20 mL) and starch indicator (1 mL) in 250 mL conical flasks. Titration with a 0.005 mol/L iodine solution revealed the first permanent trace of a dark blue-black colour, indicating the starch-iodine complex formation. The amount of ascorbic acid present in the juices was indicated by stating the quantity as milligrams of ascorbic acid per 100 mL of the sample.

#### 2.7.2. Antioxidant Activity

The antioxidant activity (AOA) of samples (lapsi juice) was evaluated using the DPPH (2,2-diphenyl-1-picrylhydrazyl) scavenging method [25]. A measured volume of the juice sample (2 mL) was combined with 2 mL of a DPPH solution (0.2 mM in ethanol), and this resulting mixture was placed in a dark environment at room temperature (25 ± 1 °C) for 30 min. A similar procedure was employed for the blank, but instead of the sample, ethanol was used. Subsequently, the absorbance values of the samples were determined at 517 nm using a UV spectrophotometer. The DPPH radical scavenging activity was calculated as a percentage (%) using the following Formula (1):

DPPH radical scavenging activity (%) = (A_o_ − A_1_)/A_o_ (1)

where A_o_ = absorbance of the control, and A_1_ = absorbance of the juice.

#### 2.7.3. Total Phenolic Content

The determination of the total phenolic compounds present in both treated and untreated fruit juices was conducted using the method suggested by Nayak et al. [26]. LJ was mixed with 0.1 mL of diluted Folin–Ciocalteu reagent, followed by a 3 min incubation. Then, 0.3 mL of 20 g/L sodium carbonate solution was added. After 2 h, absorbance was measured at 760 nm using a spectrophotometer. The findings of the trials were presented as milligrams of gallic acid equivalent (GAE) per mL of juice.

#### 2.7.4. Total Flavonoid Content

The total flavonoid content (TFC) of lapsi juice samples was determined using the approach laid out by Nayak et al. [22]. A 6 mL LJ sample was mixed with 0.2 mL of a 10% *w*/*v* aluminium chloride and 5% sodium potassium tartrate solution. After adding 5.6 mL of distilled water, the solution was incubated in the dark for 30 min. Absorbance at 415 nm was measured using a spectrophotometer, and a calibration curve was generated using catechin as the standard. The concentration of flavonoids in the lapsi juices was denoted as milligrams of catechin equivalent (CE) per mL of juice.

### 2.8. Determination of Microbial Attributes

Microbiological determination was performed to determine the total plate counts as well as total yeast and mould counts (YMC) of lapsi juices by the method followed in the work of Tomadoni et al. [27]. For total plate count, juice samples were spread on plate-count agar and incubated at 37 ± 1 °C for 48 h. Similarly, YMC was determined by spread-plating samples on potato dextrose agar and incubating at 37 ± 1 °C for 72 h. Colonies were counted after the completion of incubation periods and the results were expressed as log cfu/mL.

### 2.9. Experimental Design for Optimisation

#### Artificial Neural Network Modelling

Using MATLAB R2020a software, artificial neural networks were created to generate predictive analytics for examining the physicochemical, functional, and microbial characteristics of LJ samples that underwent thermosonication. Preliminary studies were used to designate the input parameters and their ranges. The independent variables included thermosonication amplitude X1 (50%, 75%, and 100%), thermosonication temperature X2 (30, 40, and 50 °C), and treatment time X3 (15, 30, 45, and 60 min). The output parameters considered in this study were pH, TSS, TA, cloudiness, browning index, ascorbic acid, antioxidant activity, TPC, TFC, and YMC. The 36 experimental data points were randomly divided into three sets for training (75% of the data), validation (15% of the data), and testing (15% of the data) purposes. The structure of the well-trained ANN model with the optimal topology was (3:10:10), where the weights of connections between neurons in different layers and the bias or threshold values of hidden- and output-layer neurons were organised in matrix form with a network-type feed-forward backprop along with a training function (TRAINLM). Specifically, the matrix size of the weights of synaptic connections between input- and hidden-layer neurons was 10 × 3, and the matrix size of the weights of connections between hidden- and output-layer neurons was 10 × 10. Finally, the matrix size of the bias or threshold values of hidden- and output-layer neurons was 10 × 1 and 10 × 1, respectively. The adaption function was set at LEARNGDM with transfer function PURELIN. Preliminary experiments were conducted with treatment temperatures ranging from 30 °C to 50 °C, which were determined by referring to previous studies that reported the extraction of different fruit juices using ultrasonication [28,29]. The fitness of ANN models was evaluated using coefficient of determination (R^2^), mean absolute error (MAE), and mean square error (MSE) values, which were determined using Equations (1)–(3) [29].

(2) $$R^{2}=\frac{\sum_{i=1}^{n}\left(x_{i}-\bar{x})(y_{i}-\bar{y}\right)}{\sqrt{\sum_{i=1}^{n}{\left(x_{i}-\bar{x}\right)}^{2}}\;\;\sqrt{\sum_{i=1}^{n}{\left(y_{i}-\bar{y}\right)}^{2}}}$$

(3) $$MAE=\left[\frac{1}{n}\sum_{i=1}^{n}\left(x_{i}-y_{i}\right)\right]$$

(4) $$MSE=\frac{1}{n}\sum_{i=1}^{n}(x_{i}-y_{i}{)}^{2}$$

The variables used in this context are $x_{i}$ for experimental data, $y_{i}$ for predicted values, *x* representing the mean value of experimental data, *y* representing the mean value of predicted data, and n representing the total number of observations.

R-squared (*R*^2^) is a statistical measure that represents the proportion of the variance in the dependent variable (the variables to predict) that is explained by the independent variables (the inputs to model). In the context of evaluating ANN models, *R*^2^ is often used to assess how well the model captures the variation in the target variable. It ranges from 0 to 1, with higher values indicating a better fit. An R^2^ value of 1 means the model perfectly predicts the target variable, while an *R*^2^ of 0 means the model provides no improvement over a simple mean prediction. MAE is a common metric used to measure the average absolute difference between the actual values and the predicted values. It quantifies the average magnitude of errors in the predictions made by the model. Smaller MAE values indicate better accuracy, as they imply that the model’s predictions are closer to the actual values on average. MSE is another common metric used to measure the average squared difference between the actual values and the predicted values. It squares the errors, giving more weight to larger errors. Like MAE, a lower MSE indicates better accuracy, with values closer to zero implying better model performance.

### 2.10. Statistical Analysis

All experimental analyses were conducted in triplicate, and the results were presented as mean ± standard deviation. To assess the statistical significance, the data were subjected to analysis of variance (ANOVA) using SPSS version 16.0, with a predetermined significance level of *p* < 0.05.

## 3. Results and Discussion

### 3.1. Impact of Processing on Physicochemical Properties of Lapsi Juices

#### 3.1.1. pH, TSS, and TA

The study aimed to improve the shelf life of lapsi juice by conducting thermosonication treatments under appropriate experimental conditions. In this study, the quality parameters of freshly extracted lapsi juice were compared with those of thermally and non-thermally processed juice using thermosonication. The experimental data for the pH, TSS, and TA of raw, pasteurised, and thermosonically processed lapsi juices are shown in Table 1 and Table 2.

The pH of the control sample differed significantly from that of the PSLJ and TSLJ samples. The pH values of the control and PSLJ were determined to be 3.77 ± 0.00 and 3.64 ± 0.01, respectively. Minor variations in pH were observed in the TSLJ, with values ranging from 3.32 ± 0.01 to 3.57 ± 0.01 at 50% amplitude, 3.29 ± 0.00 to 3.55 ± 0.01 at 75% amplitude, and 3.21 ± 0.02 to 3.51 ± 0.01 at 100% amplitude. The pH decrease observed in the TSLJ could be due to the generation of new chemical compounds, such as limonoids, flavonoids, carotenoids, and coumarins, resulting from the high-amplitude energy produced during ultrasonication. Nadeem et al. [30] suggested that thermosonication induces chemical reactions in aqueous environments, and because lapsi fruit contains high moisture content, the process may lead to the production of new compounds.

The total soluble solid (TSS) content of both treated and fresh lapsi juice samples did not exhibit major significant variations, with all the samples having a TSS of 11.00 ± 0.10 °Brix (Table 1 and Table 2). These findings are consistent with previous studies on juice samples that have undergone ultrasonication, which also did not result in any changes in TSS [31].

Untreated and thermally treated lapsi juices both had titratable acidities (TA) of 3.30 ± 0.01% and 3.27 ± 0.01%, respectively (Table 1 and Table 2). According to the findings, fresh and PSLJ samples had higher TA values than TSLJ samples. The TSLJ samples had TA values that ranged from 3.25 ± 0.01% to 2.88 ± 0.01%. The sonication process’s lower energy levels may not be sufficient to change the structure of larger compounds, which could account for the decrease in TA values as seen in the TSLJ samples. The amount of TPC and the generation of chemical compounds during TS may be related to the observed variations in TA levels between TSLJ and other juices. Previous studies by Basumatary et al. [11] and Lan et al. [32], reported similar findings. In conclusion, the findings demonstrate that thermosonication had a significant impact on pH and TA values but had no discernible impact on the TSS of lapsi juice.

#### 3.1.2. Cloudiness and Browning Index of Lapsi Juice

The cloudiness of fruit juices is influenced by various compounds such as cellulose, hemicellulose, pectin, and protein, and can affect their flavour and colour [33]. No significant differences (*p* < 0.05) in cloudiness values were observed between the fresh and thermally treated juice samples, as indicated in Table 1 and Table 2. The cloudiness value for the fresh samples was 0.796 ± 0.002, while the cloudiness value for the thermally treated samples was 0.794 ± 0.003. However, the CI value of TSLJ samples was significantly (*p* < 0.05) higher than that of the fresh and PSLJ samples, ranging from 0.897 ± 0.005 to 1.757 ± 0.001 after the thermosonication treatments. The CI values of lapsi juices subjected to thermosonication at 50% amplitude ranged from 0.897 ± 0.005 to 1.583 ± 0.002, while the juices treated at 75% amplitude showed cloudiness values ranging from 1.157 ± 0.001 to 1.696 ± 0.002. Additionally, lapsi juices treated at 100% amplitude showed cloudiness values ranging from 1.196 ± 0.002 to 1.757 ± 0.001. The study by Oladunjoye et al. [34] found that the amplitude, temperature, and treatment time of the TS process had a significant impact on the CI of juice samples. Cavitation-induced high-pressure-region gradients during ultrasonication may be responsible for the increased cloudiness because the process may cause macromolecules to break down into smaller ones [34]. Additionally, juices may become cloudier as a result of sonication treatments that homogenise the juices, break down larger molecules, increase the number of suspended particles, and reduce the distance between them by expanding the surface area of the liquid. The disintegration of fruit cell walls brought on by heat and ultrasound can release pectin and other compounds that give rise to the cloudiness of juice. The stability of suspended particles and cloudiness can be impacted by the presence of polysaccharides, proteins, or other substances. When suspended particles in juice are subjected to mechanical stress with ultrasonic amplitudes, their size may increase, resulting in increased cloudiness. Higher amplitudes can cause particles to expand in size. The juice becomes more turbid as a result of the light scattering caused by these larger structures. Similar results were observed in several investigations [11,26].

Table 1 and Table 2 display the browning index (BI) values for untreated and treated lapsi juice samples that underwent thermosonication and pasteurisation treatments. The results indicated that the BI values of treated juice samples were significantly different (*p* < 0.05) from those of the raw lapsi juice. The control and TSLJ samples had BI values of 0.118 ± 0.001 and 0.192 ± 0.001, respectively, while the TSLJ samples displayed BI values in the range of 0.184 ± 0.001 to 0.301 ± 0.002. The BI values of TSLJ showed a range from 0.897 ± 0.005 to 1.583 ± 0.002 at 50% amplitude, while the BI values for lapsi juices treated at 75% amplitude ranged from 0.198 ± 0.001 to 0.289 ± 0.002, and from 0.216 ± 0.001 to 0.301 ± 0.002 for 100% amplitude. Ultrasonication causes cell-wall breakdown in fruit juices, releasing enzymes such as polyphenol oxidase and peroxidase. These enzymes react with the phenolic compounds in juice to produce brown pigments. According to previous findings by Kesavan et al. [12] and Nayak et al. [22], the increase in the browning index value of treated juice samples may be due to Maillard reactions and the breakdown of colouring pigments during TS treatments. Higher amplitude ultrasonication may necessitate a longer exposure time to achieve the desired level of sonication, increasing the browning index of the juice. When ultrasonication is used on juices, it can cause physical damage to the pigments that give the juice its colour, such as chlorophyll and carotenoids. The ultrasonic waves’ high amplitude can break down the complex structures of these pigments, making them more susceptible to browning reactions [11].

### 3.2. Impact of Processing on Functional Properties of Lapsi Juices

#### 3.2.1. Ascorbic Acid

Ascorbic acid (AA) is a vitamin that is essential to produce carnitine, neurotransmitters, and collagen. It is primarily found in fruits and vegetables. It has several beneficial health effects due to its antiatherogenic, antioxidant, anti-carcinogenic, and immunomodulatory properties.

**Figure 2:**
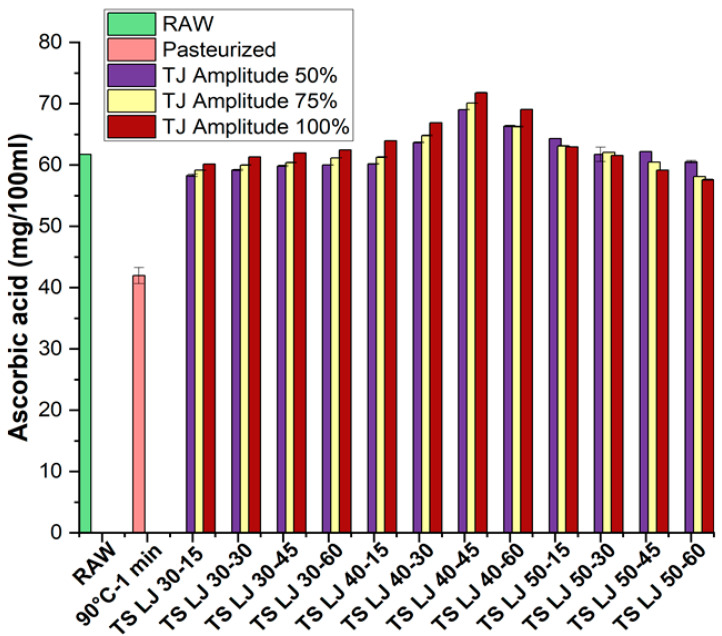
Effects of pasteurisation and thermosonication (TS) (50, 75, and 100% amplitude) on ascorbic acid levels of lapsi juice. Raw, untreated sample; 90-1, pasteurised LJ (lapsi juice); TJ, thermosonicated juice; 30-15, TS LJ at 30 °C for 15 min; 30-30, TS LJ at 30 °C for 30 min; 30-45, TS LJ at 30 °C for 45 min; 30-60, TS LJ at 30 °C for 60 min; 40-15, TS LJ at 40 °C for 15 min; 40-30, TS LJ at 40 °C for 30 min; 40-45, TS LJ at 40 °C for 45 min; 40-60, TS LJ at 40 °C for 60 min; 50-15, TS LJ at 50 °C for 15 min; 50-30, TS LJ at 50 °C for 30 min; 50-45, TS LJ at 50 °C for 45 min; 50-60, TS LJ at 50 °C for 60 min.

The AA content of untreated juice and PSLJ was measured as 61.73 ± 0.02 mg/100 mL and 41.96 ± 1.31 mg/100 mL, respectively. Figure 2 shows that the AA levels in the pasteurised juice were lower than those in the fresh lapsi juice, but the accumulation of AA was higher in the TSLJ samples (ranging from 57.59 ± 0.10 to 71.80 ± 0.06 mg/100 mL) than in the PJ. The AA content of PSLJ was lower (41.96 ± 1.31 mg/100 mL) than that of both treated and raw juice samples. The results for raw, PSLJ, and TSLJ samples are presented in Figure 2. The maximum retention of AA content obtained was 71.80 ± 0.06 (TSLJ) at 100% amplitude and 40 °C temperature for 45 min of treatment time. The decrease in AA levels in the TSLJ when compared to those in raw juices could be attributed to the sensitivity of AA to heat and the highly water-soluble nature of lapsi juices. Furthermore, the use of mild/low temperatures during sonication can be credited for the retention of AA in TSLJ as compared to that in untreated lapsi juices. This is due to AA’s inability to withstand the higher temperatures frequently used during PS [35]. It should be considered that AA is a volatile compound that degrades quickly at higher processing temperatures. As such, it preserves better at lower treating temperatures [36]. The enhancements in the retention of AA content of TSLJ may also be attributed to cell-wall breakdown, which leads to ascorbic acid liberation into the juice during TS treatment; regardless, some loss of AA may occur due to the process of oxidation [37]. Moreover, the depletion of O_2_ that is dissolved during cavitation could be correlated to a rise in AA levels, and studies have shown an improved retention of AA in fruit juice samples during TS [33,38].

#### 3.2.2. Antioxidant Activity

**Figure 3:**
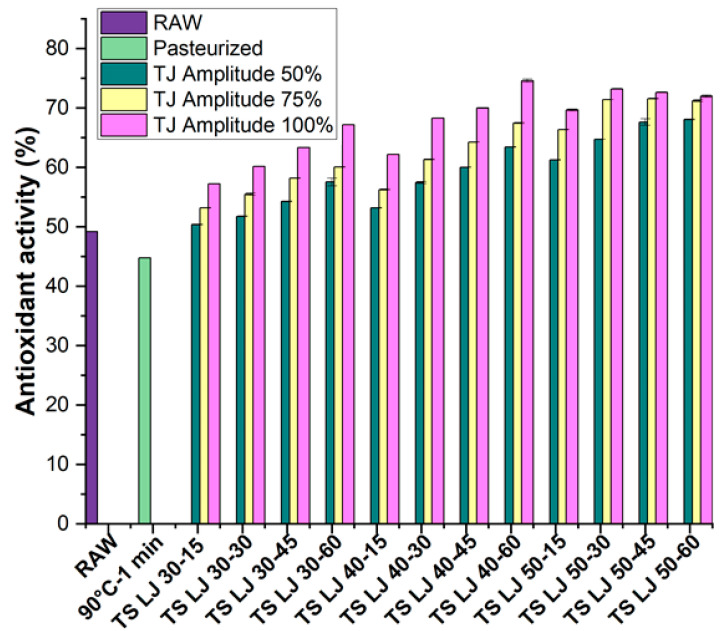
Effects of pasteurisation and thermosonication (50, 75, and 100% amplitude) on antioxidant activity levels of lapsi juice. For sample codes, refer to Figure 2.

The effects of TS and PS on the antioxidant activity (AOA) of lapsi juice samples, both fresh and treated, are represented in Figure 3. The AOA of raw and PSLJ was observed to be 49.21 ± 0.04% and 44.77 ± 0.06%, respectively. The application of excessive heat during the preparation of lapsi juices, along with the depletion of vitamin C and some carotenoids, may be responsible for a reduction in AOA of pasteurised juices.

On the other hand, the AOA of TSLJ was observed to have higher retention than that of fresh and PSLJ samples, as observed in Figure 3. Increasing the treatment amplitude and time was found to increase the AOA of lapsi juices. Specifically, TSLJ treated at 40 °C for 60 min with an amplitude of 100% exhibited a greater AOA (74.60 ± 0.28%) when compared with other TSLJ samples. The increase in the total phenolic content (TPC) of TSLJ may be responsible for the upsurge in AOA. The presence of hydroxyl groups produced by sonication in the aromatic structure of phenolic compounds may be responsible for the increase in the TPC of lapsi juices. Furthermore, it has been discovered that the presence of second hydroxyl radicals in the ortho/para positions raises the AOA of phenolic compounds. This happens because the aromatic ring’s electron density is increased, making it more susceptible to resonance stabilisation and reducing the phenolic compound’s reactivity to free radicals [39]. According to previous investigations, the execution of cavitation during thermosonication has aided in the separation and accessibility of the phenolic molecules, ultimately increasing in AOA content of TSLJ [40,41]. Studies have indicated that an increase in temperature within the range of 40–50 °C can enhance the extraction of phenolic compounds by promoting solute dispersion and diffusion. However, it should be noted that temperatures beyond 60 °C may cause denaturation of the phenolic compounds. The implementation of TS has previously been reported for improving AOA in different fruit juice samples, for example pomelo fruit [11] and tomato [40] juices.

#### 3.2.3. Total Phenolic Content

**Figure 4:**
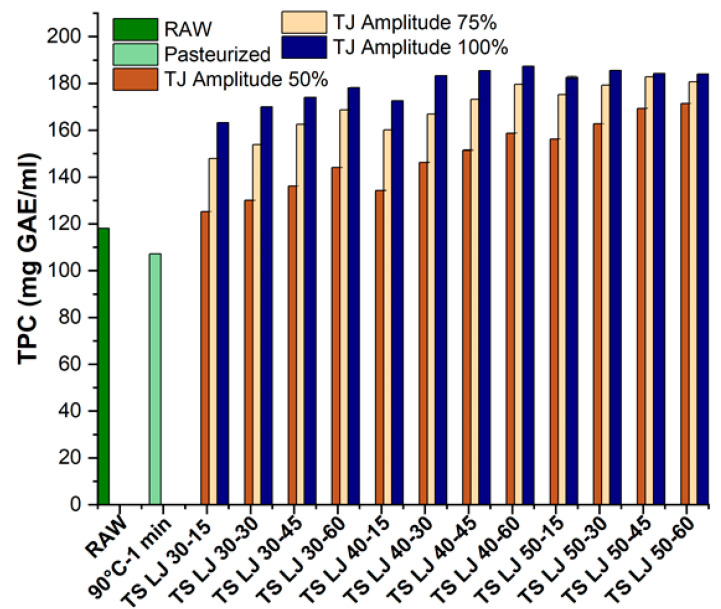
Effects of pasteurisation and thermosonication (50, 75, and 100% amplitude) on the total phenolic content of lapsi juice. For sample codes, refer to Figure 2. TPC, total phenolic content; GAE, gallic acid equivalent.

The findings of the experimental results from the TPC determination of TSLJ, PSLJ and fresh lapsi juices are represented in Figure 4. The study showed that the TPC of raw juice was greater than that of PS juice. Specifically, the total phenol levels of untreated and PSLJ samples were 118.13 ± 0.02 and 107.18 ± 0.05 mg GAE/mL, respectively. However, it was found that thermosonication significantly increased the total phenolic content (*p* < 0.05) at all three amplitudes (50, 75, and 100%). The maximum retention of phenolic content obtained was 187.33 ± 0.03 mg GAE/mL (TSLJ) at 100% amplitude and 40 °C temperature for 60 min of treatment.

The study shows that the TPC of TSLJ increased compared to that of raw and PSLJ samples, as observed in Figure 4. Furthermore, as previously stated by an investigator [42], an upsurge in TPC after TS is possibly a result of the removal of confined molecules of O_2_ from the lapsi juice samples. In addition, treatment temperature possessed a significant (*p* < 0.05) impact on the total amount of phenols (at each amplitude), and the TPC of lapsi juices increased with amplitude and temperature.

The rise in the TPC of TLJ may be credited to several factors. Initially, cavitation may necessitate phenolic compounds to change from restricted to free-form states [43]. Additionally, disintegration of cell walls due to cavitation during TS may liberate phenolic compounds and contribute to the increase in TPC [44]. Further, the microcavity formation during TS may also enhance mass transfer and contribute to the increase in TPC, as observed in previous studies by Abid et al. [31] and Nayak et al. [26].

#### 3.2.4. Total Flavonoid Content

**Figure 5:**
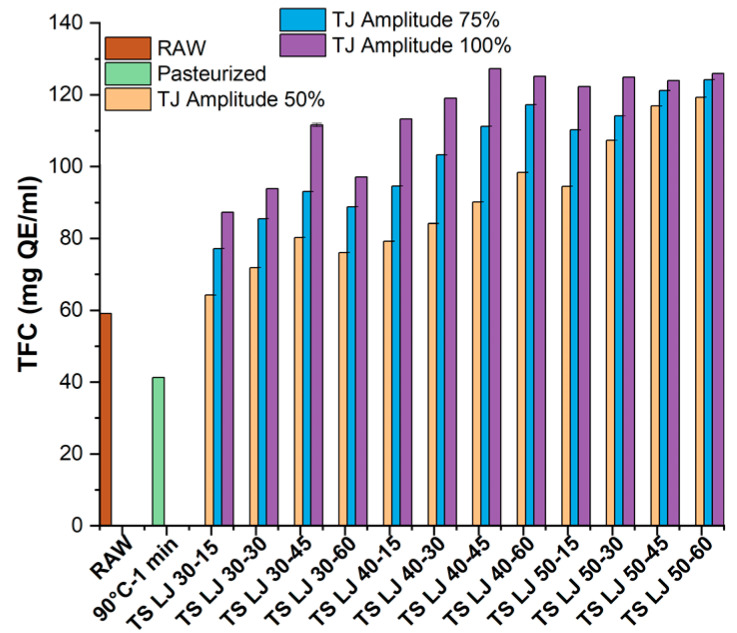
Effects of pasteurisation and thermosonication (50, 75, and 100% amplitude) on the total flavonoid content of lapsi juice. For sample codes, refer to Figure 2. TFC, total flavonoid content; QE, quercetin equivalent.

The influence of PS and TS on the TFC of lapsi juices was investigated in this study, and the results of the experiments were presented in Figure 5. The TFC of fresh and PSLJ samples was recorded as 59.07 ± 0.01 and 41.30 ± 0.08 mg QE/mL, respectively, as shown in Figure 5. The TFC values for TSLJ were found to be greater than those of control and PSLJ samples (ranging from 64.20 ± 0.05 to 127.27 ± 0.05 mg QE/mL). The treatment at amplitude 100% and temperature 40 °C for 45 min resulted in the highest flavonoid content (127.27 ± 0.05 mg QE/mL), followed by 75% amplitude at temperature 50 °C for 60 min of treatment (124.20 ± 0.02 mg QE/mL) and 50% amplitude at temperature 50 °C for 60 min of treatment (119.30 ± 0.03 mg QE/mL).

Phenolic and flavonoid substances are typically present in plant cells in bound or soluble forms. Ultrasonication can induce the release of phenolic compounds because of the cavitation generated around the particles. The overall extraction efficiency rises as a result of the high amplitude of ultrasonic waves, which improves the diffusion of flavonoids from the plant material into the juice. The flavonoid compounds’ stability and preservation are probably enhanced by the process’s precise temperature and time parameters, which stop them from degrading or denaturing. Moreover, the increase in TPC and TFC levels may be attributed to the incorporation of a hydroxyl group into the aromatic phenolic ring structure as a result of thermosonication [26]. Several researchers have reported similar findings involving an upsurge in the levels of TFC in TSLJ [11,44].

### 3.3. Impact of Processing on the Microbial Counts of Lapsi Juices

**Figure 6:**
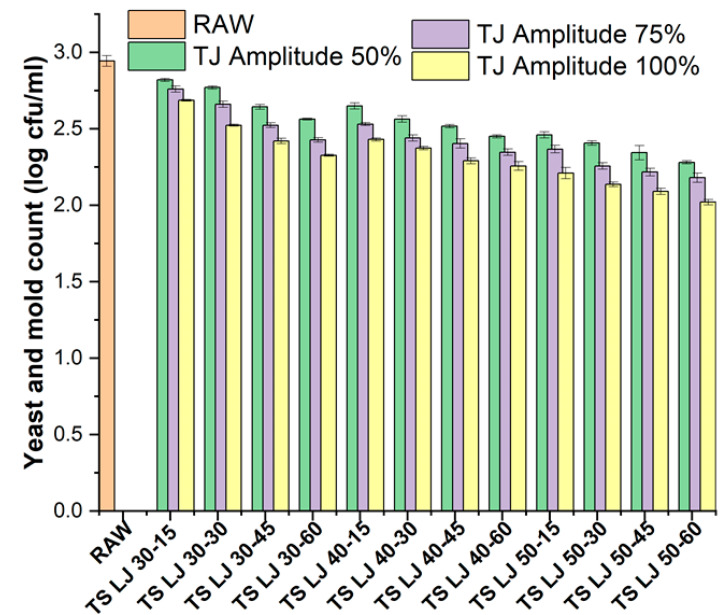
Effects of pasteurisation and thermosonication on yeast and mould counts of lapsi juice. For sample codes, refer to Figure 2. cfu, colony forming unit.

The effect of TS on microbial counts is presented in Figure 6. Prior to any treatment, the raw juice displayed total plate count and YMC values of 2.44 ± 0.03 and 2.94 ± 0.04 log cfu/mL, respectively. PSLJ treatment completely inactivated microbial populations (not detectable [ND]) as well as yeast and mould populations (ND), which is consistent with previous research on various fruit juices [42].

The high temperature of pasteurisation causes damage to the cell membrane, nucleic acids, and protein structure of the microorganisms present in the juice. This damage to the microorganisms can cause cytolytic reactions, which eventually kill them. The heat causes the microorganisms’ cell membranes to become unstable and rupture, resulting in the leakage of the contents from cells [45]. Figure 6 shows the effects of PS and TS on the YMC of lapsi juice. The results from the TS experiments also showed complete bacterial population inactivation, as observed by the total plate count data, which was found to be ND. Bacterial populations are rendered completely inactive during TS, which combines the use of heat and ultrasound. There may be a mix of chemical and physical mechanisms at work in this phenomenon. The cavitation bubbles, which are produced by the rapid expansion and contraction of ultrasound waves in the liquid medium, are one of the physical mechanisms. These bubbles produce shock waves and high pressures that can harm the bacteria’s cell walls and membranes and render them inactive. In addition to physical mechanisms, TS can also inactivate bacteria through chemical processes like the production of free radicals. The bacterial cells may become damaged and inactive as a result of the free radicals produced by the cavitation. Furthermore, since heat can cause denaturation and degradation of bacterial proteins, nucleic acids, and other cellular components, the temperature rise that occurs during TS may also help to inactivate bacteria.

The YMC of the TS-treated juice was lower than the YMC of the fresh juice samples (2.94 ± 0.04 log cfu/mL), ranging from 2.02 ± 0.02 to 2.82 ± 0.01 log cfu/mL. However, unlike the TPC, the YMC was not completely eliminated, which could be attributed to yeast and mould’s higher resistance to sonication compared to that of bacterial populations, as previously reported [42]. A decrease in YMC is caused by an increase in either amplitude or temperature. This implies that higher levels of amplitude and temperature have a greater effect on reducing YMC than lower levels of these parameters. Yeast and mould counts can be reduced for a variety of reasons by using thermosonication with a high amplitude, temperature, and time. One possible explanation is the physical effect of cavitation, which can damage the cell membrane and cause cell death. Another reason is the generation of free radicals, which can lead to oxidative stress and cell damage. Furthermore, an increase in temperature can cause protein denaturation, which can result in microorganism inactivation. The combination of these effects can result in a reduction in yeast and mould counts in treated juice [31]. To produce juices with the least amount of nutritional and sensory loss, additional antimicrobials or high-pressure recuperation might be required in addition to thermosonication.

### 3.4. The Efficiency and Evaluation of the Established ANN Model

**Figure 7:**
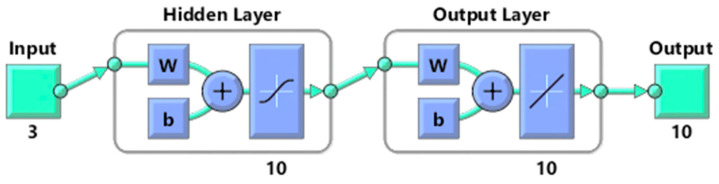
The topology of a well-developed ANN model for TSLJ. Under the input, 3 indicates the independent parameters (amplitude, temperature, and time of TS treatment), while output 10 indicates the dependent parameters (pH, TSS, TA, CI, BI, AA, AOA, TPC, TFC, and YMC). W, weight; b, bias; ANN, artificial neural network; TSLJ, thermosonicated lapsi juice; TSS, total soluble solid; TA, titratable acidity; CI, cloudiness index; BI, browning index; AA, ascorbic acid; AOA, antioxidant activity; TPC, total phenolic content; TFC, total flavonoid content; YMC, yeast and mould count.

The optimal parameters for the artificial neural network (ANN) were determined using various criteria such as a correlation plot, mean absolute error, correlation coefficient, and performance plot, as described in previous studies [16]. After testing different network topologies, a network with a hidden layer of ten neurons was found to provide the best results. The structure of the well-trained ANN model with the optimal topology (3:10:10) is presented in Figure 7, where the connection weights between neurons in different layers and the bias/ tipping-point values of neuron layers (hidden and output) are organised in the form of a matrix. Specifically, the acreage of the matrix of the neuronal connection weights among neurons of layers (input and hidden) was 10 × 3, and the acreage of matrix of the connection weights between neurons of the hidden and output layers was 10 × 10. Finally, the size of matrix of the bias/ tipping-point values of neurons (hidden and output layers) was 10 × 1 and 10 × 1, correspondingly.

**Figure 8:**
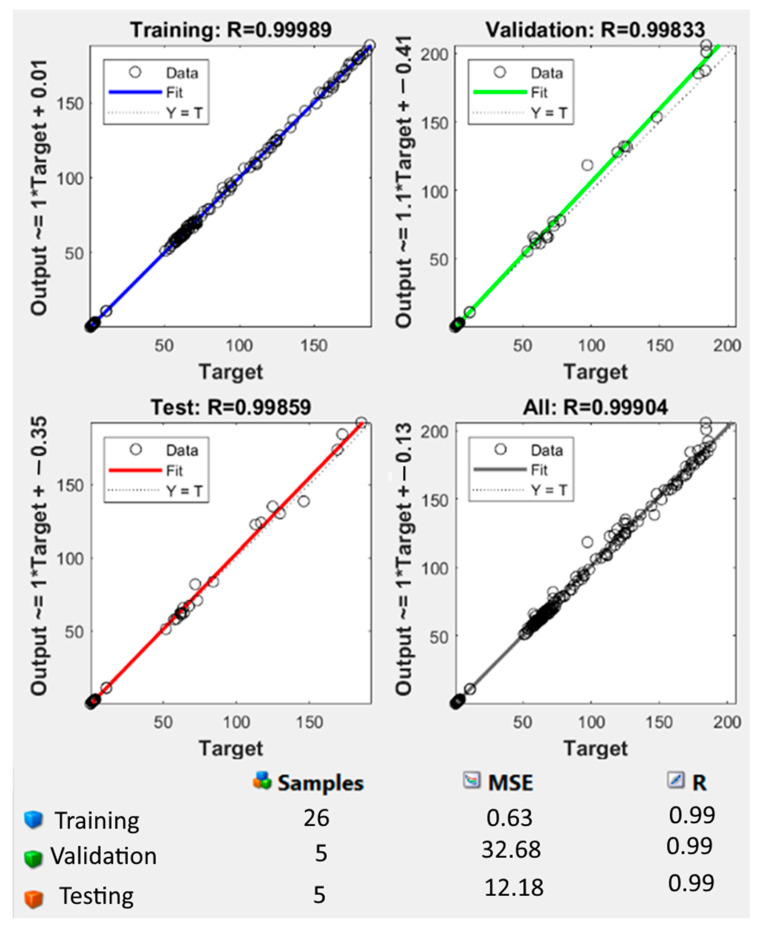
The correlation coefficient (R) of training, test, and all data of the well-developed ANN model. MSE, mean square error; Output, predicted data generated by the ANN; Target, expected outcome for the given input; Training, adjustment in internal parameters (weights and biases) based on a labelled dataset; Testing, accuracy, precision, recall, and mean square error, depending on the type of task (classification or regression); Validation, monitoring and fine-tuning of the model’s performance; ANN, artificial neural network.

**Figure 9:**
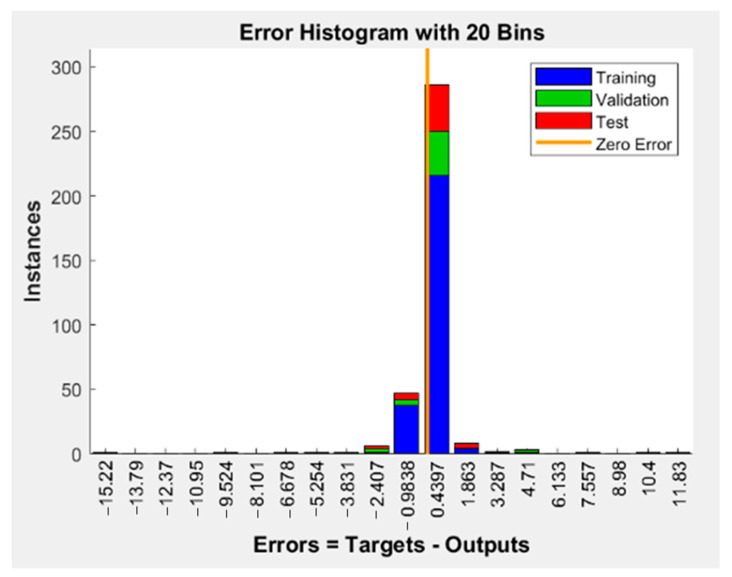
Error histogram of the developed ANN model. Instances, the number of data points or examples that fall into different error or loss bins within the histogram; Zero error, model’s predictions matching the true target values exactly for those instances; ANN, artificial neural network.

The performance of the generated ANN model was assessed using the correlation coefficient (R), which was found to be 1.0 for the training set and 0.99989 for the test data set. R was 0.99904, as observed in Figure 8. The variance between the target or found value of the response and the resultant value of the established ANN model was calculated using the error histogram, as shown in Figure 9. The histogram’s minimum bar at zero error showed that the established ANN model was extremely accurate [13]. In addition to the correlation coefficient, statistical parameters such as the coefficient of determination (R^2^), MSE, and mean absolute error (MAE) were calculated to verify the developed ANN model [16]. Table 3 shows the experimental output and Table 4 displays the R^2^, MSE, and MAE values for all responses obtained from the ANN modelling of lapsi juice processing by thermosonication [16]. The R^2^ for pH, TSS, TA, CI, BI, AA, AOA, TPC, TFC, and YM values were 0.97, 0.91, 0.99, 0.99, 0.99, 0.99, 0.97, 0.99, 0.99, and 0.99, respectively. All values are satisfactory and acceptable according to the literature [46]. A minimum value of 0.80 for R^2^ is considered a criterion for the best-fit model [28]. The developed ANN model’s R^2^ values exceeded 0.80 for all responses, demonstrating the model’s viability [13]. Table 4 reports the anticipated values of the responses obtained from the established ANN model.

**Figure 10:**
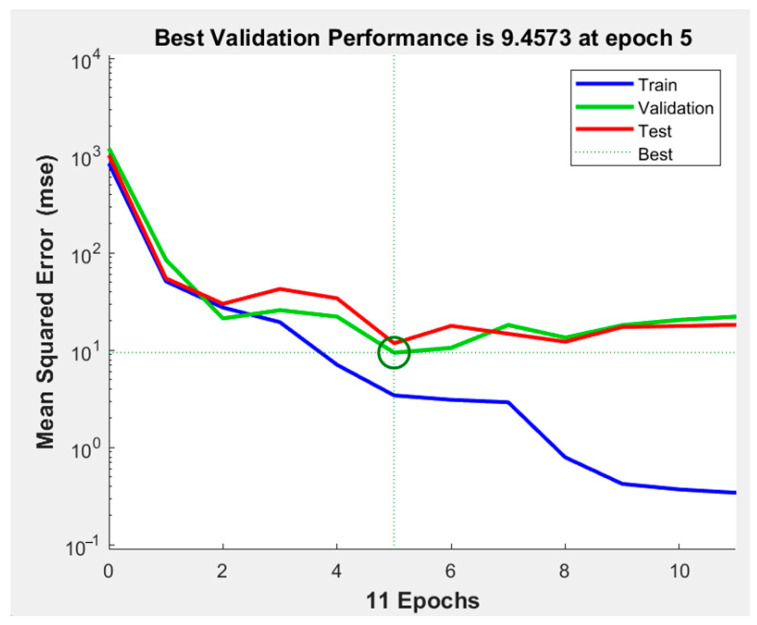
The performance of MSE with respect to an increase in the number of the epochs. Epochs: the number of epochs is a hyperparameter that needs to be set by the user. Epochs: number of time iterations of the entire training dataset. MSE, mean square error. The green circle represents in the context of an ANN’s MSE performance, indicating a specific data point is converging to a desired value.

Three sets of the 36 experimental observations were selected at random for training (75% of the experimental data), validation (15% of the experimental data), and testing (15% of the experimental data) purposes, respectively. The network parameters were established using the training process, validated to evaluate the network parameters’ stability, and tested for the management of network parameter error [46]. In this study, a neural network model with input (three), hidden (ten), and output (ten) layers was used, as shown in Figure 7. To develop the ideal ANN model for predicting responses, it was necessary to estimate the neuron numbers within the hidden layer by continually training the entire network until a minimum MSE and maximum R was achieved. Based on the results, the hidden layer’s number of neurons was set to ten, which showed that the minimum MSE for pH, TSS, TA, CI, BI, AA, AOA, TPC, TFC, and YM were 0.42, 0.17, 0.77, 0.47, 0.82, 0.10, 0.21, 0.25, 0.96, and 0.13 for the training, testing, validation, and all sets of data, respectively. Figure 8 and Figure 9 show the ANN schematic’s post-training effectiveness, an error histogram, and analysis of regression. The performance of MSE for training, validation, and testing data as a function of the number of epochs is presented in Figure 10. The plot shows that the network performance improves as the number of epochs increases, and the best authentication performance was at 5 epochs, with an MSE reading of 9.4573. According to the erroneous value histogram in Figure 8, numerous information points have an error value of 0.4390, indicating that the model performs well in predicting the target values. Figure 8 depicts the results of the regression test, which reveals a significant relationship between the anticipated results and the real target values for all data sets. Table 4 shows the predicted values of the responses obtained using the ANN model, and there is good match between the actual and predicted values, as shown in Table 3 and Table 4. This strong correlation between actual and predicted values confirms the model’s stability. This model’s successful performance is significant because it illustrates how useful it could be as a forecasting tool for future experiments. When conducting experiments, time and resources can be saved by using the ANN model to predict the response for a specific set of input parameters. The strong correlation between the actual and predicted values also boosts confidence in the data’s dependability and the precision of the experiment’s findings. Despite the ANN model’s encouraging results, it is essential to remain aware that the model must still be validated using more experimental data. This may aid in enhancing the model’s accuracy and dependability even more. Overall, the accuracy with which the ANN model was able to predict the response is a positive development for the field of food processing and may pave the way for more effective and efficient processing methods [13,16,28].

### 3.5. ANN-Based Process for Parameter Optimisation

ANN is a widely used approach that employs various functions such as crossover, choices, and alteration of entities in the populace in order to optimise non-linear and near-linear issues [13,16]. The ideal conditions for processing lapsi juice with thermosonication have been identified through the use of the ANN and are listed after 20 iterations: At treatment condition 1 (50% amplitude, 50 °C, and 60 min), 2 (75% amplitude, 50 °C, and 45 min), 3 (75% amplitude, 40 °C. and 45 min), 4 (75% amplitude, 50 °C, and 60 min), 5 (100% amplitude, 40 °C, and 45 min), 6 (100% amplitude, 40 °C, and 60 min), and 7 (100% amplitude, 50 °C, and 60 min). Table 5 presents the physicochemical, functional, and microbial attributes obtained from the optimised treatment combination, which resulted in the highest and most desirable values. These values showed a satisfactory match with the predicted values, proving the ANN model’s accuracy and capacity for improvement. Significant differences between raw and pasteurised juice parameters were also found, demonstrating the value of the ideal treatment settings [28].

## 4. Conclusions

The current investigation used a multi-layer ANN to model and optimise the ultrasound-assisted extraction of lapsi juice. The influence of thermosonication at various levels of amplitude, temperature, and time on various responses such as physicochemical (pH, TSS, TA, CI, and BI), nutritional (AA, AOA, TPC, and TFC), and microbiological (total plate count and YMC) properties was also ascertained. The maximum levels of AA (71.80 ± 0.05 mg/100 mL), AOA (74.60 ± 0.28%), TPC (187.33 ± 0.03 mg GAE/mL), and TFC (127.27 ± 0.05 mg QE/mL) were obtained. The microbial populations in TS samples were recorded as lower than those in fresh samples and were attained by total plate count (not detected) and YMC (2.02 ± 0.02 log cfu/mL) in TSLJ under optimal TS conditions. For AA and TFC, the optimal conditions were 100% amplitude, 40 °C, and 45 min. For AOA and TPC, the optimal conditions were 100% amplitude, 40 °C, and 60 min, and for YMC, the optimal conditions were 100% amplitude, 50 °C, and 60 min. The high R values indicated a high degree of concurrence among the actual and predicted values. The optimal extraction variables have been identified using an established ANN as follows: The ideal conditions for processing lapsi juice with thermosonication have been identified through ANN and are listed following 20 iterations. The actual responses at the optimised process parameters closely matched the predicted values acquired from the established ANN model. In accordance with the outcomes, thermosonication might serve as an intriguing alternative to thermal pasteurisation for preserving lapsi juice while minimising or avoiding quality deterioration and augmenting functional attributes.

### Industrial Relevance

Ultrasound-assisted extraction (UAE) is an innovative method that is gaining popularity in a variety of industries, including the food and beverage industry. When it comes to the extraction of lapsi fruit juice, UAE provides several benefits and has significant industrial significance. UAE not only improves extraction efficiency and speed, but it also improves the quality of lapsi fruit juice. The moderate and non-thermal nature of ultrasonic waves reduces the breakdown of heat-sensitive components found in fruit, such as vitamins, enzymes, and antioxidants. As a result, the extracted juice maintains its nutritious value, brilliant colour, and flavour profile. This element of UAE quality preservation is very important for the food and beverage industry, as customers are increasingly looking for natural and less processed products. It is the best option for the food and beverage industry because of its adaptability in terms of process parameters and its ability to improve extraction efficiency, speed, and quality. UAE represents a viable technique for industrial-scale lapsi fruit juice extraction, providing firms with a competitive edge in the market with the added advantages of sustainability and environmental concern.

## Figures and Tables

**Table 1:** Quality parameters of raw and pasteurised lapsi fruit juice ^1^.

Parameter	Raw Juice	Pasteurised Juice
pH	3.77 ± 0.00 ^a^	3.64 ± 0.01 ^b^
TSS (°Brix)	11 ± 0.00 ^a^	11 ± 0.00 ^a^
TA (%)	3.30 ± 0.01 ^a^	3.27 ± 0.01 ^b^
Cloudiness	0.796 ± 0.002 ^a^	0.794 ± 0.003 ^a^
Browning index	0.118 ± 0.001 ^a^	0.192 ± 0.001 ^b^
Ascorbic acid (mg/100 mL)	61.73 ± 0.02 ^a^	41.96 ± 1.31 ^b^
Antioxidant activity (%)	49.21 ± 0.04 ^a^	44.77 ± 0.06 ^b^
TPC (mg GAE/mL)	118.13 ± 0.02 ^a^	107.18 ± 0.05 ^b^
TFC (mg QE/mL)	59.07 ± 0.01 ^a^	41.30 ± 0.08 ^b^
Total plate count (log cfu/mL)	2.44 ± 0.03	ND
Yeast and mould count (log cfu/mL)	2.94 ± 0.04	ND

^1^ Data are presented as mean ± standard deviation. Mean values with different lowercase superscript alphabets in the same row (a,b) are significantly different (*p* < 0.05) from each other. ND, not detected; GAE, gallic acid equivalents; QE, quercetin equivalent; TA, titratable acidity; TFC, total flavonoid content; TPC, total phenolic content; TSS, total soluble solids; cfu, colony forming unit.

**Table 2:** Impact of thermosonication at 50, 75, and 100% amplitude on pH, TSS, TA, cloudiness, and browning index of lapsi juice ^1^.

Parameter	Amplitude (%)	15 min			30 min			45 min			60 min		
		30 °C	40 °C	50 °C	30 °C	40 °C	50 °C	30 °C	40 °C	50 °C	30 °C	40 °C	50 °C
pH	50	3.57 ± 0.01 ^aA^	3.53 ± 0.01 ^bA^	3.48 ± 0.01 ^dA^	3.55 ± 0.01 ^aA^	3.47 ± 0.00 ^dA^	3.39 ± 0.01 ^eA^	3.50 ± 0.01 ^cA^	3.38± 0.00 ^eA^	3.33 ± 0.01 ^fgA^	3.47 ± 0.01 ^dA^	3.35 ± 0.01 ^fA^	3.32 ± 0.01 ^gA^
	75	3.55 ± 0.01 ^aA^	3.48 ± 0.01 ^bhA^	3.45 ± 0.00 ^cA^	3.51 ± 0.01 ^dA^	3.40 ± 0.01 ^eA^	3.33 ± 0.01 ^fiA^	3.48 ± 0.01 ^bA^	3.35 ± 0.00 ^fA^	3.29 ± 0.00 ^gA^	3.46 ± 0.01 ^hcA^	3.33 ± 0.01 ^iA^	3.30 ± 0.01 ^gA^
	100	3.51 ± 0.01^aB^	3.43 ± 0.01 ^bB^	3.39 ± 0.01 ^cB^	3.47 ± 0.00 ^dB^	3.38 ± 0.01 ^cB^	3.27 ± 0.01 ^eB^	3.44 ± 0.01 ^bB^	3.31 ± 0.01 ^fB^	3.22 ± 0.01 ^gB^	3.40 ± 0.01 ^cB^	3.27 ± 0.01 ^eB^	3.21 ± 0.02 ^gB^
TSS (°Brix)	50	10.93 ± 0.06 ^aA^	11.03 ± 0.06 ^aA^	11 ± 0.00 ^aA^	11.03± 0.06 ^aA^	11 ± 0.10 ^aA^	11.00 ± 0.10 ^aA^	10.67 ± 0.58 ^aA^	11.00± 0.10 ^aA^	10.93 ± 0.12 ^aA^	11.13 ± 0.12 ^aA^	10.70 ± 0.61 ^aA^	11.03 ± 0.06 ^aA^
	75	11 ± 0.10 ^aA^	11.03 ± 0.15 ^aA^	11.03 ± 0.06 ^aA^	11.03 ± 0.06 ^aA^	11 ± 0.00 ^aA^	11.10 ± 0.10 ^aA^	11.00 ± 0.10 ^aA^	11.03 ± 0.06 ^aA^	11.00 ± 0.00 ^aA^	10.90 ± 0.10 ^aA^	11.00 ± 0.00 ^aA^	11.03 ± 0.06 ^aA^
	100	11.03 ± 0.06 ^aA^	11 ± 0.10 ^aA^	11.03 ± 0.06 ^aA^	10.97 ± 0.12 ^aA^	10.93 ± 0.12 ^aA^	10.97 ± 0.06 ^aA^	11.00 ± 0.00 ^aA^	10.93 ± 0.06 ^aA^	10.97 ± 0.06 ^aA^	10.93 ± 0.12 ^aA^	11.00 ± 0.00 ^aA^	10.93 ± 0.12 ^aA^
TA (%)	50	3.25 ± 0.01 ^aA^	3.17 ± 0.01 ^cA^	3.12 ± 0.01 ^eA^	3.21 ± 0.01 ^bA^	3.14 ± 0.02 ^deA^	3.09 ± 0.01 ^fA^	3.16 ± 0.01 ^cdA^	3.12 ± 0.01 ^eA^	3.03 ± 0.01 ^gA^	3.15 ± 0.01 ^cdA^	3.08 ± 0.01 ^fA^	3.01 ± 0.01 ^gA^
	75	3.19 ± 0.02 ^aA^	3.16 ± 0.01 ^abA^	3.08 ± 0.01 ^efA^	3.17 ± 0.01 ^abA^	3.10 ± 0.02 ^deA^	3.03 ± 0.01 ^ghA^	3.14 ± 0.01 ^bcA^	3.09 ± 0.00 ^eA^	3.02 ± 0.01 ^ghA^	3.13 ± 0.02 ^cdA^	3.05 ± 0.01 ^fgA^	3.01 ± 0.01 ^hA^
	100	3.14 ± 0.02 ^aB^	3.12 ± 0.01 ^aB^	3.03 ± 0.01 ^cB^	3.12 ± 0.01 ^aB^	3.07 ± 0.01 ^bB^	2.94 ± 0.01^eB^	3.08 ± 0.01 ^bB^	3.00 ± 0.02 ^dB^	2.90 ± 0.01 ^fB^	3.05 ± 0.01 ^bcB^	2.97 ± 0.01 ^eB^	2.88 ± 0.01 ^fB^
CI	50	0.89 ± 0.00 ^aA^	1.10 ± 0.01 ^bA^	1.29 ± 0.00 ^cA^	1.02 ± 0.00 ^dA^	1.26 ± 0.02 ^eA^	1.38 ± 0.00 ^fA^	1.17 ± 0.00 ^gA^	1.37 ± 0.00 ^fA^	1.49 ± 0.00 ^hA^	1.26 ± 0.00 ^eA^	1.40 ± 0.00 ^iA^	1.58 ± 0.01 ^jA^
	75	1.20 ± 0.00 ^aB^	1.40 ± 0.01 ^bB^	1.60 ± 0.00 ^cB^	1.29 ± 0.00 ^dB^	1.47 ± 0.00 ^eB^	1.62 ± 0.00 ^fB^	1.37 ± 0.00 ^gB^	1.52 ± 0.00 ^hB^	1.65 ± 0.00 ^iB^	1.43 ± 0.00^jB^	1.59 ± 0.00 ^kB^	1.69 ± 0.01 ^lB^
	100	1.20 ± 0.00 ^aB^	1.41 ± 0.01 ^bB^	1.63 ± 0.01 ^cB^	1.32 ± 0.00 ^dB^	1.50 ± 0.00 ^eB^	1.67 ± 0.00 ^fB^	1.40 ± 0.00 ^gB^	1.58 ± 0.00 ^hB^	1.70 ± 0.00 ^iB^	1.49 ± 0.00 ^jB^	1.61 ± 0.00 ^kB^	1.75 ± 0.01 ^lB^
BI	50	0.20 ± 0.00 ^aA^	0.18 ± 0.01 ^aA^	0.20 ± 0.01 ^bA^	0.18 ± 0.00 ^caA^	0.19 ± 0.00 ^dA^	0.22 ± 0.00 ^eA^	0.19± 0.00 ^daA^	0.20 ± 0.00 ^bA^	0.23 ± 0.001 ^fA^	0.19 ± 0.00 ^bA^	0.21 ± 0.00 ^gA^	0.26 ± 0.01 ^hA^
	75	0.20 ± 0.00 ^aB^	0.22 ± 0.01 ^bB^	0.27 ± 0.01 ^cB^	0.21 ± 0.00 ^dB^	0.24 ± 0.00 ^eB^	0.28 ± 0.00 ^fB^	0.22 ± 0.00 ^bB^	0.25 ± 0.00 ^gB^	0.28 ± 0.00 ^fjB^	0.23 ± 0.00 ^hB^	0.26 ± 0.00 ^iB^	0.29 ± 0.01 ^jB^
	100	0.21 ± 0.00 ^aC^	0.24 ± 0.01 ^bC^	0.28 ± 0.01 ^cC^	0.23 ± 0.00 ^dC^	0.26 ± 0.00 ^eC^	0.29 ± 0.00 ^fjC^	0.24 ± 0.00 ^bC^	0.27 ± 0.00 ^gC^	0.30 ± 0.00 ^hC^	0.25 ± 0.00 ^iC^	0.29 ± 0.00 ^jC^	0.30 ± 0.01 ^fhC^

^1^ Data are presented as mean ± standard deviation. Mean values with different uppercase superscript (A–C) in the same column and lowercase (a–l) in the same row are significantly different (*p* < 0.05) from each other. TA, titratable acidity; TSS, total soluble solid; CI, cloudiness; BI, browning index.

**Table 3:** Experimental outputs for the conducted thermosonication trials ^1^.

Treatment	pH	TSS	TA	CI	BI	AA	AOA	TPC	TFC	YM
TSLJ-50-30-15	3.57	10.93	3.25	0.897	0.184	58.31	50.37	125.22	64.20	2.82
TSLJ-50-30-30	3.55	11.03	3.21	1.023	0.187	59.18	51.76	130.05	71.86	2.77
TSLJ-50-30-45	3.50	10.67	3.16	1.174	0.190	59.85	54.28	136.15	80.27	2.64
TSLJ-50-30-60	3.47	11.13	3.15	1.268	0.197	60.00	57.54	144.05	76.07	2.56
TSLJ-50-40-15	3.53	11.03	3.17	1.091	0.185	60.17	53.18	134.23	79.21	2.65
TSLJ-50-40-30	3.47	11.00	3.14	1.263	0.193	63.67	57.45	146.24	84.17	2.56
TSLJ-50-40-45	3.38	11.00	3.12	1.373	0.200	69.03	60.02	151.45	90.17	2.52
TSLJ-50-40-60	3.35	10.70	3.08	1.408	0.211	66.34	63.42	158.76	98.36	2.45
TSLJ-50-50-15	3.48	11.00	3.12	1.295	0.199	64.33	61.25	156.27	94.53	2.46
TSLJ-50-50-30	3.39	11.00	3.09	1.384	0.222	61.72	64.73	162.76	107.32	2.41
TSLJ-50-50-45	3.33	10.93	3.03	1.494	0.233	62.18	67.63	169.30	116.86	2.34
TSLJ-50-50-60	3.32	11.03	3.01	1.583	0.258	60.53	68.08	171.43	119.30	2.28
TSLJ-75-30-15	3.55	11.00	3.19	1.157	0.198	59.13	53.18	147.85	77.16	2.76
TSLJ-75-30-30	3.51	11.03	3.17	1.291	0.212	59.98	55.47	153.86	85.48	2.66
TSLJ-75-30-45	3.48	11.00	3.14	1.375	0.225	60.39	58.21	162.49	93.05	2.52
TSLJ-75-30-60	3.46	10.90	3.13	1.436	0.236	61.16	60.07	168.73	88.81	2.43
TSLJ-75-40-15	3.48	11.03	3.16	1.383	0.228	61.28	56.27	160.13	94.57	2.53
TSLJ-75-40-30	3.40	11.00	3.10	1.472	0.244	64.81	61.34	166.89	103.25	2.44
TSLJ-75-40-45	3.35	11.03	3.09	1.524	0.252	70.09	64.26	173.24	111.23	2.40
TSLJ-75-40-60	3.33	11.00	3.05	1.596	0.261	66.26	67.46	179.63	117.25	2.35
TSLJ-75-50-15	3.45	11.03	3.08	1.603	0.277	63.11	66.35	175.29	110.22	2.37
TSLJ-75-50-30	3.33	11.10	3.03	1.626	0.283	62.05	71.42	179.28	114.13	2.26
TSLJ-75-50-45	3.29	11.00	3.02	1.657	0.287	60.47	71.57	182.79	121.19	2.22
TSLJ-75-50-60	3.30	11.03	3.01	1.696	0.289	58.13	71.23	180.70	124.20	2.18
TSLJ-100-30-15	3.51	11.03	3.14	1.196	0.216	60.15	57.25	163.18	87.31	2.69
TSLJ-100-30-30	3.47	10.97	3.12	1.322	0.232	61.33	60.16	169.94	93.84	2.52
TSLJ-100-30-45	3.44	11.00	3.08	1.401	0.242	61.95	63.31	174.04	111.60	2.42
TSLJ-100-30-60	3.40	10.93	3.05	1.494	0.254	62.44	67.17	178.23	97.07	2.33
TSLJ-100-40-15	3.43	11.00	3.12	1.411	0.244	63.93	62.20	172.63	113.25	2.43
TSLJ-100-40-30	3.38	10.93	3.07	1.502	0.262	66.88	68.28	183.31	119.05	2.37
TSLJ-100-40-45	3.31	10.93	3.00	1.589	0.277	71.80	69.99	185.40	127.27	2.29
TSLJ-100-40-60	3.27	11.00	2.97	1.613	0.295	69.06	74.60	187.33	125.16	2.26
TSLJ-100-50-15	3.39	11.03	3.03	1.636	0.282	62.95	69.66	182.54	122.26	2.21
TSLJ-100-50-30	3.27	10.97	2.94	1.675	0.297	61.57	73.20	185.59	124.93	2.14
TSLJ-100-50-45	3.22	10.97	2.90	1.707	0.302	59.13	72.63	184.20	123.94	2.09
TSLJ-100-50-60	3.21	10.93	2.88	1.757	0.301	57.59	71.98	184.00	125.90	2.02

^1^ TSLJ, thermosonicated lapsi juice at amplitude 50, 75, and 100%; temperature 30, 40, and 50 °C; and treatment time 15, 30, 45, and 60 min. TSS, total soluble solid; TA, titratable acidity; CI, cloudiness index; BI, browning index; AA, ascorbic acid; AOA, antioxidant activity; TPC, total phenolic content; TFC, total flavonoid content; YM, yeast and mould count.

**Table 4:** Predicted network outputs from ANN modelling ^1^.

Treatment	pH	TSS	TA	CI	BI	AA	AOA	TPC	TFC	YM
TSLJ-50-30-15	3.55	10.97	3.22	0.958	0.203	58.31	51.63	129.22	68.23	2.82
TSLJ-50-30-30	3.54	11.00	3.21	1.029	0.204	59.23	52.51	132.12	71.99	2.77
TSLJ-50-30-45	3.52	11.00	3.14	1.259	0.204	59.91	52.49	136.69	82.69	2.64
TSLJ-50-30-60	3.39	10.97	3.02	1.382	0.200	59.43	56.26	148.36	75.54	2.56
TSLJ-50-40-15	3.54	11.01	3.20	1.052	0.233	63.61	53.74	134.11	75.57	2.66
TSLJ-50-40-30	3.47	11.03	3.13	1.262	0.226	64.98	56.69	143.83	84.04	2.56
TSLJ-50-40-45	3.35	10.99	3.04	1.456	0.213	67.83	56.03	149.34	100.89	2.52
TSLJ-50-40-60	3.35	10.98	3.03	1.493	0.221	65.89	60.98	158.93	96.69	2.51
TSLJ-50-50-15	3.45	11.02	3.11	1.265	0.210	64.74	60.68	148.93	88.29	2.46
TSLJ-50-50-30	3.36	11.00	3.06	1.385	0.230	61.76	66.50	162.30	99.41	2.40
TSLJ-50-50-45	3.32	10.98	3.03	1.407	0.264	61.89	67.07	168.72	116.72	2.35
TSLJ-50-50-60	3.32	10.98	3.02	1.451	0.266	60.61	69.60	172.78	117.94	2.28
TSLJ-75-30-15	3.55	11.02	3.20	0.964	0.244	59.48	54.06	144.90	76.51	2.76
TSLJ-75-30-30	3.50	11.00	3.16	1.124	0.235	60.28	57.32	157.25	86.04	2.66
TSLJ-75-30-45	3.48	11.00	3.15	1.367	0.231	60.90	57.80	161.98	93.16	2.57
TSLJ-75-30-60	3.41	11.00	3.07	1.551	0.206	60.18	59.45	165.06	84.34	2.43
TSLJ-75-40-15	3.50	11.05	3.18	1.187	0.225	62.80	58.41	159.43	92.58	2.53
TSLJ-75-40-30	3.42	11.03	3.13	1.537	0.203	65.84	61.90	167.54	99.56	2.55
TSLJ-75-40-45	3.34	11.00	3.05	1.670	0.190	69.85	63.45	172.89	110.06	2.40
TSLJ-75-40-60	3.30	10.98	2.97	1.689	0.188	66.53	65.99	177.42	113.05	2.33
TSLJ-75-50-15	3.37	11.05	3.07	1.470	0.193	63.98	66.16	175.13	108.38	2.37
TSLJ-75-50-30	3.35	11.04	3.04	1.575	0.190	61.67	70.50	180.54	115.91	2.26
TSLJ-75-50-45	3.32	11.00	3.01	1.558	0.201	60.20	70.96	180.89	120.18	2.22
TSLJ-75-50-60	3.27	10.93	2.95	1.517	0.233	58.38	69.85	178.88	121.57	2.14
TSLJ-100-30-15	3.49	10.99	3.15	0.991	0.253	60.94	57.72	164.01	87.20	2.69
TSLJ-100-30-30	3.46	10.98	3.13	1.233	0.261	62.26	60.47	169.09	95.38	2.52
TSLJ-100-30-45	3.44	10.99	3.10	1.422	0.256	63.62	63.35	173.72	98.28	2.42
TSLJ-100-30-60	3.43	11.00	3.08	1.565	0.230	63.66	64.88	178.03	99.63	2.33
TSLJ-100-40-15	3.40	10.96	3.07	1.295	0.273	64.58	63.05	174.28	112.16	2.43
TSLJ-100-40-30	3.37	10.95	3.04	1.563	0.250	71.29	68.20	181.98	120.78	2.42
TSLJ-100-40-45	3.33	10.96	3.00	1.626	0.226	71.47	71.01	184.89	124.04	2.34
TSLJ-100-40-60	3.30	10.99	2.95	1.635	0.217	69.29	70.42	185.21	124.91	2.26
TSLJ-100-50-15	3.36	11.00	3.02	1.602	0.197	62.70	69.97	182.86	122.66	2.21
TSLJ-100-50-30	3.29	10.98	2.96	1.627	0.193	61.03	71.70	184.04	124.04	2.14
TSLJ-100-50-45	3.27	10.96	2.94	1.635	0.191	58.87	72.38	184.32	124.61	2.09
TSLJ-100-50-60	3.25	10.95	2.92	1.633	0.190	57.79	71.12	183.31	125.48	2.02
R²	0.978	0.912	0.996	0.999	0.996	0.999	0.977	0.999	0.990	0.998
MSE	0.426	0.173	0.775	0.478	0.825	0.101	0.214	0.253	0.961	0.136
MAE	0.006	−0.013	0.012	0.000	0.020	−0.412	0.352	0.394	0.998	−0.007

^1^ TSLJ, thermosonicated lapsi juice 50, 75 and 100%, temperature 30, 40 and 50 °C, and treatment time 15, 30, 45 and 60 min. TSS, total soluble solid; TA, titratable acidity; CI, cloudiness; BI, browning index; AA, ascorbic acid; AOA, antioxidant activity; TPC, total phenolic content; TFC, total flavonoid content; YM, yeast and mould count; R, coefficient of determination; MSE, mean square error; MAE, mean absolute error, ANN, artificial neural network.

**Table 5:** Comparison of optimised treatment conditions obtained using ANN for TSLJ, raw juice, and PSLJ ^1^.

Treatment	pH	TSS	TA	CI	BI	AA	AO	TPC	TFC	YM
RLJ	3.77 ± 0.00 ^a^	11 ± 0.00 ^a^	3.29 ± 0.01 ^a^	0.79 ± 0.00 ^a^	0.11 ± 0.00 ^a^	61.73 ± 0.02 ^a^	49.21 ± 0.04 ^a^	118.13 ± 0.02 ^a^	59.07± 0.01 ^a^	2.94 ± 0.04 ^a^
PSLJ	3.64 ± 0.01 ^b^	11 ± 0.00 ^a^	3.26 ± 0.01 ^b^	0.79 ± 0.00 ^a^	0.19 ± 0.00 ^b^	41.96 ± 1.31 ^b^	44.77 ± 0.06 ^b^	107.18 ± 0.05 ^b^	41.30 ± 0.08 ^b^	ND
TSLJ-50-50-60	3.32 ± 0.01 ^c^	11.02 ± 0.06 ^a^	3.01 ± 0.01 ^cd^	1.58 ± 0.00 ^b^	0.25 ± 0.00 ^c^	60.53 ± 0.21 ^c^	68.08 ± 0.07 ^c^	171.43 ± 0.05 ^c^	119.30 ± 0.03 ^c^	2.28 ± 0.01 ^b^
TSLJ-75-50-45	3.29 ± 0.0 ^d^	11 ± 0.00 ^a^	3.02 ± 0.01 ^d^	1.65 ± 0.00 ^c^	0.28 ± 0.00 ^d^	60.47 ± 0.03 ^c^	71.57 ± 0.06 ^d^	182.79 ± 0.12 ^d^	121.19 ± 0.06 ^d^	2.22 ± 0.03 ^ce^
TSLJ-75-40-45	3.35 ± 0.0 ^e^	11.03 ± 0.05 ^a^	3.09 ± 0.00 ^e^	1.52 ± 0.00 ^d^	0.25 ± 0.00 ^e^	70.09 ± 0.02 ^d^	64.26 ± 0.03 ^e^	173.24 ± 0.01 ^e^	111.23 ± 0.04 ^e^	2.40 ± 0.03 ^d^
TSLJ-75-50-60	3.29 ± 0.01 ^d^	11.03 ± 0.05 ^a^	3.01 ± 0.00 ^cd^	1.69 ± 0.00 ^e^	0.28 ± 0.00 ^f^	58.13 ± 0.03 ^e^	71.23 ± 0.17 ^f^	180.70 ± 0.07 ^f^	124.20 ± 0.03 ^f^	2.18 ± 0.03 ^c^
TSLJ-100-40-45	3.31 ± 0.01 ^c^	10.93 ± 0.05 ^a^	3.00 ± 0.01 ^c^	1.58 ± 0.00 ^f^	0.27 ± 0.00 ^g^	71.80 ± 0.06 ^f^	69.99 ± 0.07 ^g^	185.40 ± 0.03 ^g^	127.27 ± 0.05 ^g^	2.29 ± 0.02 ^b^
TSLJ-100-40-60	3.26 ± 0.01 ^f^	11.00 ± 0.00 ^a^	2.96 ± 0.01 ^f^	1.61 ± 0.00 ^g^	0.29 ± 0.00 ^h^	69.06 ± 0.04 ^g^	74.60 ± 0.28 ^h^	187.33 ± 0.03 ^h^	125.16 ± 0.04 ^h^	2.26 ± 0.03 ^e^
TSLJ-100-50-60	3.20 ± 0.01 ^g^	10.93 ± 0.11 ^a^	2.87 ± 0.01 ^g^	1.75 ± 0.00 ^h^	0.30 ± 0.00 ^i^	57.59 ± 0.10 ^e^	71.98 ± 0.18 ^i^	184.00 ± 0.04 ^i^	125.90 ± 0.07 ^i^	2.02 ± 0.02 ^f^

^1^ RLJ, raw lapsi juice; PSLJ, pasteurised lapsi juice; TSLJ, thermosonicated lapsi juice 50, 75, and 100%, temperature of 30, 40, and 50 °C, and treatment time of 15, 30, 45, and 60 min; TSS, total soluble solid; TA, titratable acidity; CI, cloudiness index; BI, browning index; AA, ascorbic acid; AOA, antioxidant activity; TPC, total phenolic content; TFC, total flavonoid content; YM, yeast and mould count; ND, not detected; ANN, artificial neural network. Values with different superscript lowercase alphabets in the same column (a–i) are significantly different (*p* < 0.05) from each other.

## Data Availability

The data used to support the findings of this study can be made available by the corresponding author upon request.

## Abbreviations

TA	Titratable acidity
TSS	Total soluble solid
CI	Cloudiness index
BI	Browning index
AA	Ascorbic acid
AOA	Antioxidant activity
TPC	Total phenolic content
TFC	Total flavonoid content
YMC	Yeast and mould count
R	Coefficient of determination
MSE	Mean square error
MAE	Mean absolute error
TS	Thermosonication
PS	Pasteurisation
LJ	Lapsi juice
RLJ	Raw lapsi juice
TSLJ	Thermosonicated lapsi juice
PSLJ	Pasteurised lapsi juice
ND	Not detected
ANN	Artificial neural network
GAE	Gallic acid equivalents
QE	Quercetin equivalent
cfu	Colony forming unit
